# Supplementary material for: Single Cell Transcriptomic and Chromatin Profiles Suggest Layer Vb Is the Only Layer With Shared Excitatory Cell Types in the Medial and Lateral Entorhinal Cortex
Source: Front Neural Circuits. 2022 Jan 26;15:806154. doi: 10.3389/fncir.2021.806154 (PMC8826650; doi:10.3389/fncir.2021.806154)
Supplement: Supplementary file 14 [file Data_Sheet_1.ZIP › Inh_vs_Exc_Motifs/homerResults/motif5.similar.html]

motif5

## Information for motif5

G
A
C
T
A
C
T
G
G
T
A
C
C
G
A
T
A
C
T
G
C
G
T
A
A
C
G
T
A
G
C
T
A
T
G
C
C
T
A
G
  
Reverse Opposite:  

G
A
T
C
T
A
C
G
T
C
G
A
T
G
C
A
A
C
G
T
A
G
T
C
C
G
T
A
A
C
T
G
G
T
A
C
C
T
G
A
  

|  |  |
| --- | --- |
| p-value: | 1e-58 |
| log p-value: | -1.355e+02 |
| Information Content per bp: | 1.629 |
| Number of Target Sequences with motif | 5836.0 |
| Percentage of Target Sequences with motif | 47.67% |
| Number of Background Sequences with motif | 15588.0 |
| Percentage of Background Sequences with motif | 40.41% |
| Average Position of motif in Targets | 299.8 +/- 242.3bp |
| Average Position of motif in Background | 340.6 +/- 317.0bp |
| Strand Bias (log2 ratio + to - strand density) | 0.0 |
| Multiplicity (# of sites on avg that occur together) | 1.37 |
| Motif File: | file (matrix) reverse opposite |

### Similar de novo motifs found

|  |  |  |  |  |  |  |  |
| --- | --- | --- | --- | --- | --- | --- | --- |
| Rank | Match Score | Redundant Motif | P-value | log P-value | % of Targets | % of Background | Motif file |
| 1 | 0.828 | T G C A C G T A C G A T C A G T G T A C C G A T A C T G G T A C C G A T A T C G G C T A A G C T | 1e-52 | -120.180409 | 22.21% | 16.85% | motif file (matrix) |
| 2 | 0.754 | G A T C A T C G G A T C G C A T T C A G G T C A C A G T C A G T T A C G T C G A | 1e-41 | -95.847402 | 9.81% | 6.56% | motif file (matrix) |
| 3 | 0.844 | G A C T A G T C C G T A A C G T A G T C C T G A T C A G G C A T | 1e-40 | -94.058721 | 33.96% | 28.37% | motif file (matrix) |
| 4 | 0.671 | C T G A G T A C G A C T A C T G A T G C C T A G A C T G C G T A | 1e-30 | -70.047635 | 21.72% | 17.63% | motif file (matrix) |
| 5 | 0.739 | A C T G T G A C C G A T T A C G C G T A T A C G C G A T T A G C G C T A A C T G T A G C G C T A | 1e-27 | -62.849101 | 3.29% | 1.82% | motif file (matrix) |
| 6 | 0.646 | C G T A A T G C G C A T C T A G A G T C C T G A C T A G G C A T G C T A G A C T | 1e-21 | -49.799436 | 11.98% | 9.34% | motif file (matrix) |
| 7 | 0.669 | G T A C G C T A C A G T A G T C C G T A C T A G T G A C G T C A G C T A T G C A T C G A T G C A | 1e-19 | -44.532442 | 3.40% | 2.11% | motif file (matrix) |
| 8 | 0.657 | C A G T A T C G T C G A C G A T C T A G C T G A C A T G A C G T T A G C C T G A T A G C G C T A | 1e-15 | -35.370723 | 4.72% | 3.34% | motif file (matrix) |
